# Genome-wide alteration in DNA hydroxymethylation in the sperm from bisphenol A-exposed men

**DOI:** 10.1371/journal.pone.0178535

**Published:** 2017-06-05

**Authors:** Huajun Zheng, Xiaoyu Zhou, De-kun Li, Fen Yang, Hongjie Pan, Tianqi Li, Maohua Miao, Runsheng Li, Wei Yuan

**Affiliations:** 1 Key Laboratory of Reproduction Regulation of NPFPC, SIPPR, IRD, Fudan University, Shanghai, China; 2 WHO Collaborating Center for Research in Human Reproduction, Shanghai, China; 3 Division of Research, Kaiser Foundation Research Institute, Kaiser Permanente Northern California, Oakland, and Department of Health Research and Policy, School of Medicine, Stanford University, Stanford, California, United States of America; University of Missouri Columbia, UNITED STATES

## Abstract

Environmental BPA exposure has been shown to impact human sperm concentration and motility, as well as rodent spermatogenesis. However, it is unclear whether BPA exposure is associated with alteration in DNA hydroxymethylation, a marker for epigenetic modification, in human sperm. A genome-wide DNA hydroxymethylation study was performed using sperm samples of men who were occupationally exposed to BPA. Compared with controls who had no occupational BPA exposure, the total levels of 5-hydroxymethylcytosine (5hmc) increased significantly (19.37% increase) in BPA-exposed men, with 72.69% of genome regions harboring 5hmc. A total of 9,610 differential 5hmc regions (DhMRs) were revealed in BPA-exposed men relative to controls, which were mainly located in intergenic and intron regions. These DhMRs were composed of 8,670 hyper-hMRs and 940 hypo-hMRs, affecting 2,008 genes and the repetitive elements. The hyper-hMRs affected genes were enriched in pathways associated with nervous system, development, cardiovascular diseases and signal transduction. Additionally, enrichment of 5hmc was observed in the promoters of eight maternally expressed imprinted genes in BPA-exposed sperm. Some of the BPA-affected genes, for example, *MLH1*, *CHD2*, *SPATA12* and *SPATA20* might participate in the response to DNA damage in germ cells caused by BPA. Our analysis showed that enrichment of 5hmc both in promoters and gene bodies is higher in the genes whose expression has been detected in human sperm than those whose expression is absent. Importantly, we observed that BPA exposure affected the 5hmc level in 11.4% of these genes expressed in sperm, and in 6.85% of the sperm genome. Finally, we also observed that BPA exposure tends to change the 5hmc enrichment in the genes which was previously reported to be distributed with the trimethylated Histone 3 (H3K27me3, H3K4me2 or H3K4me3) in sperm. Thus, these results suggest that BPA exposure likely interferes with gene expression via affecting DNA hydroxymethylation in a way partially dependent on trimethylation of H3 in human spermatogenesis. Our current study reveals a new mechanism by which BPA exposure reduces human sperm quality.

## Introduction

Bisphenol A (BPA) is an endocrine disrupting chemical used mainly in epoxy resin and polycarbonate plastic industry. Over 90 percent of the study subjects in the US had detectable total urinary BPA levels [1]. Human studies have shown that BPA exposure is associated with sexual function as well as sexual hormones among males [2, 3]. We identified an inverse association between total urinary BPA levels and semen concentration, total sperm count, sperm vitality and sperm motility among factory workers [4]. The inverse association was also reported in men recruited from different infertility clinics [5–7].

Male rodents exposed to various levels of BPA displayed several reproductive and developmental impacts, including decreased serum testosterone levels and sperm quality [8]. For example, BPA has been shown to impact sperm motility *in vivo* and *in vitro* [9, 10]. *In vitro* exposing sperm to BPA also impacted fertilization and early embryonic development [9]. Among the known epigenetic regulatory mechanisms, DNA methylation, via downregulating gene expression [11], has been demonstrated to play an important role during mammalian spermatogenesis [12, 13], as well as in maintenance of human sperm quality[14]. Diverse effects of BPA on different cells/tissues have been linked to abnormal pattern of DNA methylation [15–17]. For example, paternal BPA exposure of mice resulted in a global alteration of DNA methylation in offspring’s sperm which is associated with an impaired spermatogenesis [17] and heart problems[18] observed in offspring.

We previously reported that sperm LINE-1 methylation level was significantly lower in BPA-exposed workers compared to unexposed workers [19]. However, little is known regarding the mechanisms by which BPA modulates DNA methylation.

5-hydroxymethylcytosine (5hmc) is oxidized from 5-methylcytosine (5mC) by TET family of proteins, and has been recently recognized as a product in intermediate process of DNA demethylation [20, 21]. Importantly, dynamics of 5hmC has been detected during mouse spermatogenesis, and enrichment of 5hmc is associated with active gene transcription [22]. Given LINE-1 methylation has been treated as a marker of global DNA methylation, therefore the hypomethylation of LINE-1 present in sperm of BPA-exposed workers [19] is consistent with the hypothesis that BPA exposure promotes DNA hydroxymethylation in sperm in these workers. In the present study, we examined whether global level of DNA hydroxymethylation is different between BPA-exposed workers and unexposed controls.

## Results

### DNA hydroxymethylomes of human sperm

In order to obtain a general overview of 5hmC in the matured human spermatozoa and the impact of BPA exposure to adult sperm, we collected sperm samples from 26 adults as controls, and samples from 30 workers in a factory manufacturing BPA. The sperm from control and BPA-exposed men were pooled separately for 5-hMeDIP-seq. DNA pooling approach facilitated whole-genome 5hmc analysis of sperm which usually required large quantities of DNA. Compared with data generated from individual samples, the analysis based on DNA pools was validated to provide an accurate and reliable quantitative estimate of DNA methylation, showing highly significant correlations between individual samples and pools (95% bootstrapped confidence intervals: 0.94 to 0.96) [23, 24]. Pooled samples have been widely used in epigenetic studies, including genome-wide analysis of DNA methylation in human amnion, peripheral blood mononuclear cells, cell-free DNA of cancer, etc., [25–27].

#### Globally increased 5hmc levels in sperm from BPA-exposed men

We profiled the 5hmc distribution in human sperm cells. Based on the hMeDIP-seq data, we calculated the short read coverage at genome-wide 100-bp bins using ‘MEDIPS’[28] and transformed them into RPKM.

DNA 5hmc in sperm from BPA-exposed men was detected in 72.69% of genome (22,500,965 regions), while it was 60.89% of genome (18,849,185 regions) in sperm from the men without occupational BPA exposure (hereafter referred to as controls), indicating that BPA exposure significantly raises global level of DNA hydroxymethylation by 19.3% in human sperm.

**Fig 1:**
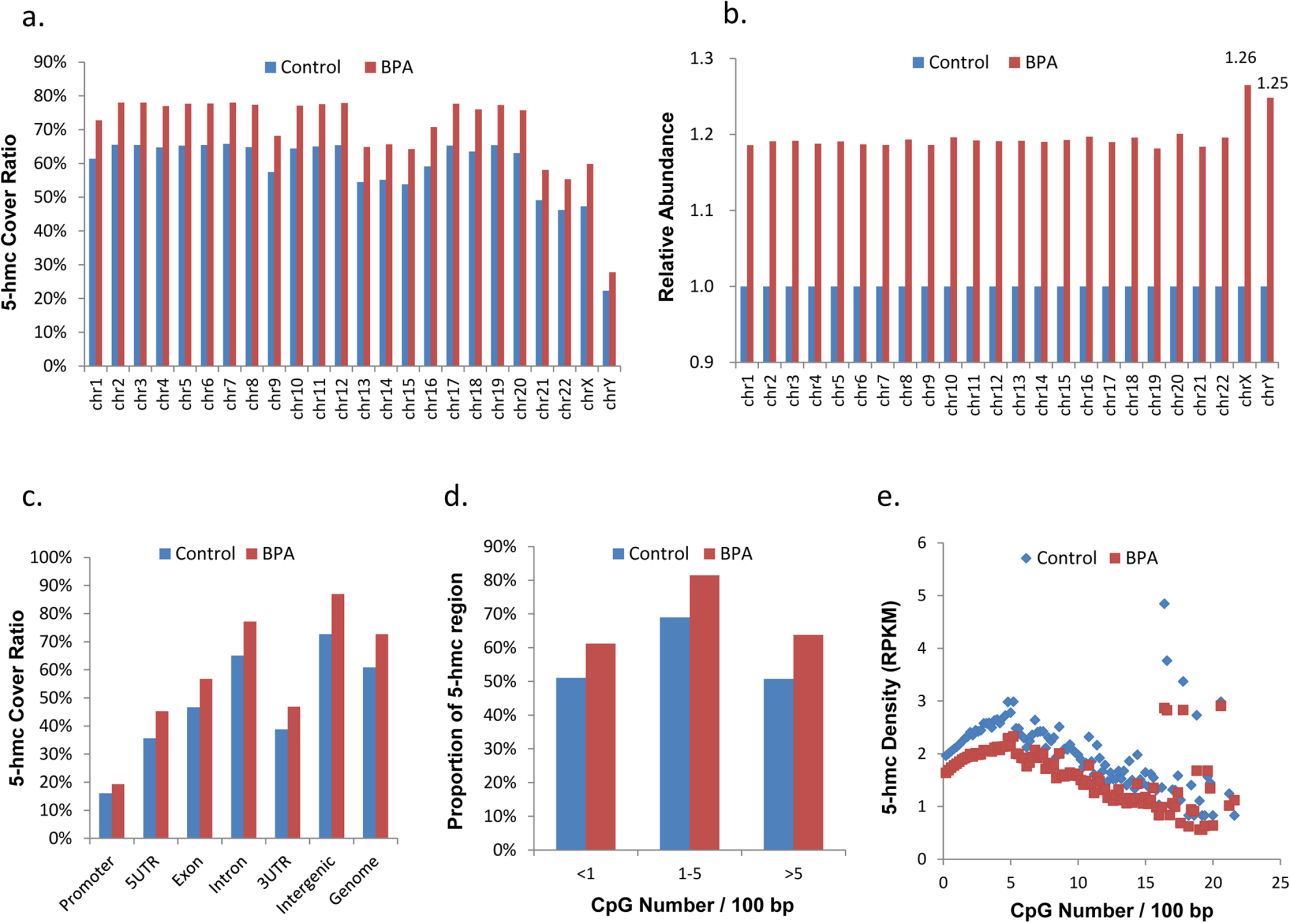
5hmc distribution in sperm genome. **(a)**. 5hmc region ratio on the 19 autosomal chromosomes and the X and Y sex chromosomes. 100% cover ratio is defined as covering all the human genome. **(b)**. 5hmc increase ratio on the 19 autosomal chromosomes and the X and Y sex chromosomes, where hMeDIP-seq data covered genome size in each chromosome was defined as 1. **(c)** The ratio of 5hmc region in each gene body. 100% cover ratio is defined as covering all of the elements of a particular type in the human genome. **(d)** Proportion of 5hmC regions with different CpG densities. **(e)** Densities of 5hmC (counts per kilobases per million reads) in each 100bp regions with different CpG densities.

We then examined the 5hmc rates on each chromosome (**Fig 1A**), and found that in both BPA-exposed and the control samples, most chromosomes exhibited high 5hmc rates (>50%), while Chromosome Y showed the lowest 5hmc level (22.72% in control group and 27.80% in BPA-exposed group). Strikingly, we observed that BPA exposure evenly increased ~19% of 5hmc level in autosomes, while in sex chromosomes, the 5hmc level increased 25–26% (**Fig 1B**), suggesting that 5hmc rates in sex chromosomes are more sensitive to BPA exposure. Relative to autosomes, the increased 5hmc in sex chromosomes showed a bit bias to 5’-UTR (15.3% in sex chromosomes vs 14.1% in autosomes) and LINE (12.6% in sex chromosomes vs 10.4% in autosomes).

The increase of 5hmc level was also reflected in each gene body (**Fig 1C** and **S1 Table**), with a maximum increase of 26% in 5’UTR region (35.64% of 5’UTR region in control and 45.26% in BPA-exposed sperm was covered by hMeDIP-seq data) and minimum increase of 18% in intron (65.1% in control and 77.19% in BPA-exposed sperm).

#### 5hmC and CpG density

A total of 28,217,009 CpGs sites are present in human genome. MeDIP-seq data of human sperm had revealed that ~60% of all CpGs in the human genome were methylated, and promoters display an inverse correlation between CpG density and methylation [29]. Since Ten-eleven translocation (Tet) family proteins can enzymatically convert 5mc to 5hmC, we postulated that 5hmc levels in the sperm genome correlated with CpG density. The hMeDIP-seq data coverage analysis shows a medium CpG coverage saturation, with 16.2 million CpGs (57.3%) in the control samples and 19.8 million CpGs (70.2%) in the BPA-exposed samples, were sequenced to a depth of at least 1x coverage (**S1 Fig**). The BPA-exposed sperm harbored about 3.6 million (or higher by 22.5%) more hydroxymethylated CpGs than the control sperm, suggesting that BPA exposure affects spermatogenesis via disturbing DNA methylation-hydroxymethylation balance.

5hmC-enriched regions were associated with moderate CpG density (1–5 CpG per 100bp) in mouse embryonic stem cells [30]. Our study exhibited a consistent result, with 68.9% of moderate CpG density region covered by hMeDIP-seq data in normal sperm, while the coverage for low CpG density region (<1 CpG per 100bp) and high CpG density region (>5 CpG per 100bp) were 51.1% and 50.7%, respectively (**Fig 1D**). In low and moderate CpG density regions, the 5hmc density increased with CpG density (**Fig 1E**); while in high CpG density regions, the 5hmc density showed an inverse correlation with CpG density.

#### 5hmc in CpG islands (CGI)

A total of 28,691 CGIs with average length of 761 bp and average number of CpG sites of 69 were observed in human genome (from UCSC Genome Browser “CpG Islands” track). Among them, 20,078 and 16,319 CGI were captured by hMeDIP-seq in BPA-exposed sperm (69.98%) and the control sperm (56.88%), respectively (**S2 Table**), indicating that the 5hmc level of CGI in sperm from BPA-exposed men is 23.0% higher than the control, in line with the result that the 5hmc level of CpGs in sperm with BPA exposure was higher by 22.5% than the control.

#### 5hmc in promoters

A total of 29,310 candidate promoter regions (2-kb-upstream of transcription start site (TSS)) of human genes were used to analyze the hydroxymethylation status of promoters. 10,993 promoters in sperm with BPA exposure (37.5%) with average RPKM of 28.5 and 10,538 in the control (35.9%) with average PRKM of 28.0 were covered by our hMeDIP data (**S3 Table**), and the difference is not statistically significant. It suggests that BPA-associated upregulated rates of 5hmc had no bias in promoter regions. In addition, 5hmc level also showed an even distribution along the whole promoter region.

### Identification of differentially hydroxymethylated regions (DhMRs)

**Fig 2:**
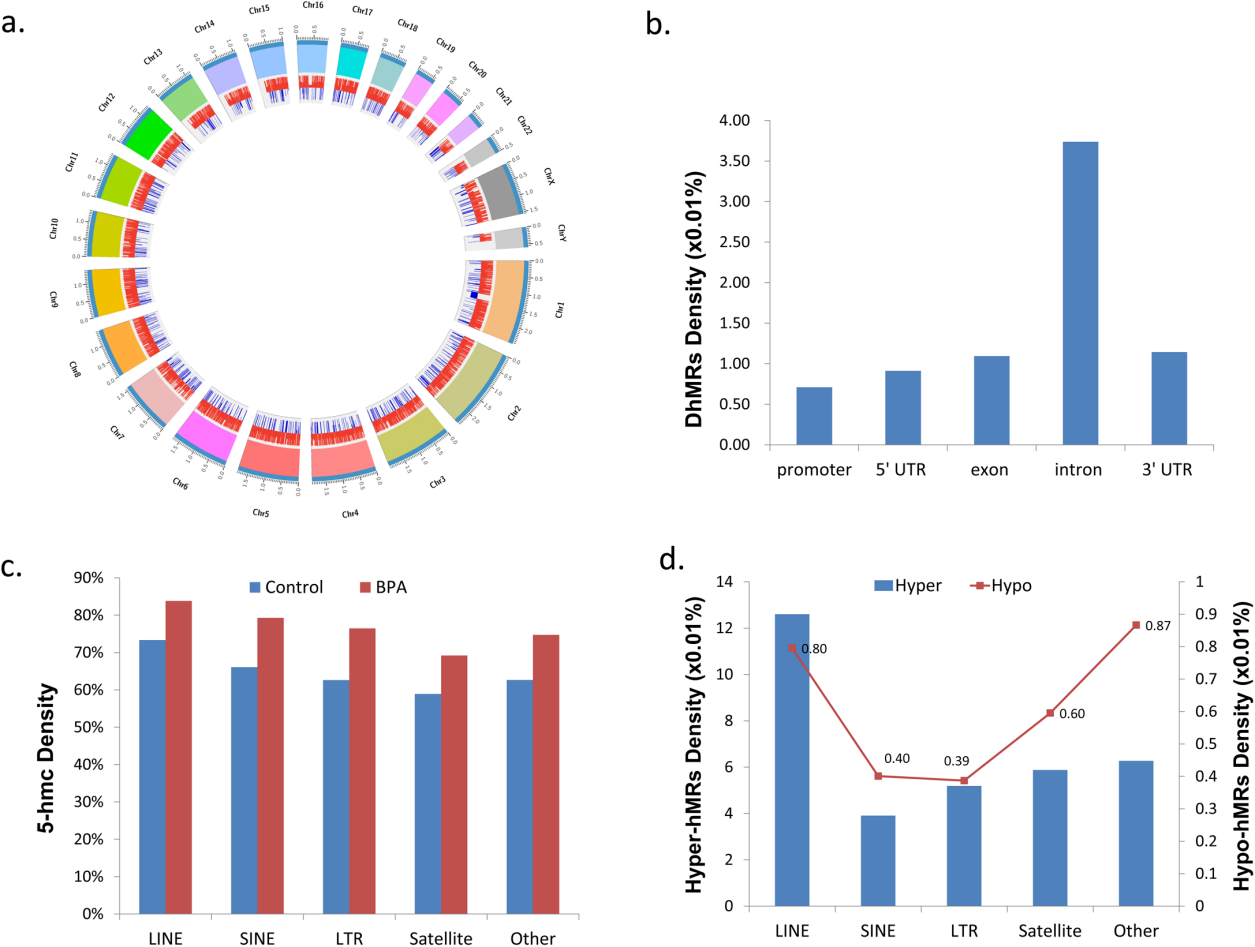
Summary of DhMR location distribution in sperm genome. **a.** Atlas of DhMRs distribution in different chromosomes. Red lines in inner circle represent the position of hyper-hMRs and blue lines represent the position of hypo-hMRs. The length unit of the outer circle is 100Mb. **b**. DhMRs in gene body. **c.** 5hmc density in repeats. **d.** DhMRs affected repeats density.

We next analyzed the hydroxymethylated regions (hMRs) whose 5hmc rates are BPA-responsive in sperm, and named them differentially 5hmc regions (DhMRs). P-values were calculated using edgeR by comparing the RPKM of the sperm with BPA exposure and control samples within each of the 100-bp windows. DhMRs were identified by filtering for windows associated with a P-value ≤0.005. This step filtered 9,610 DhMRs between sperm with BPA exposure and control, with an average RPKM 7.2 in sperm with BPA exposure and 1.8 in control (**Fig 2A** and **S4 Table**). In addition, we noticed that chromosome 19 exhibited the highest DhMRs density. Chromosome 19 is extremely gene dense relative to other human chromosomes [31], indicating that DhMRs might be associated with gene distribution. Genomic region enrichment analysis of these DhMRs indicated that they were mainly located in intron regions (**Fig 2B**).

#### Distribution of DhMRs in the repetitive sequences

**Fig 3:**
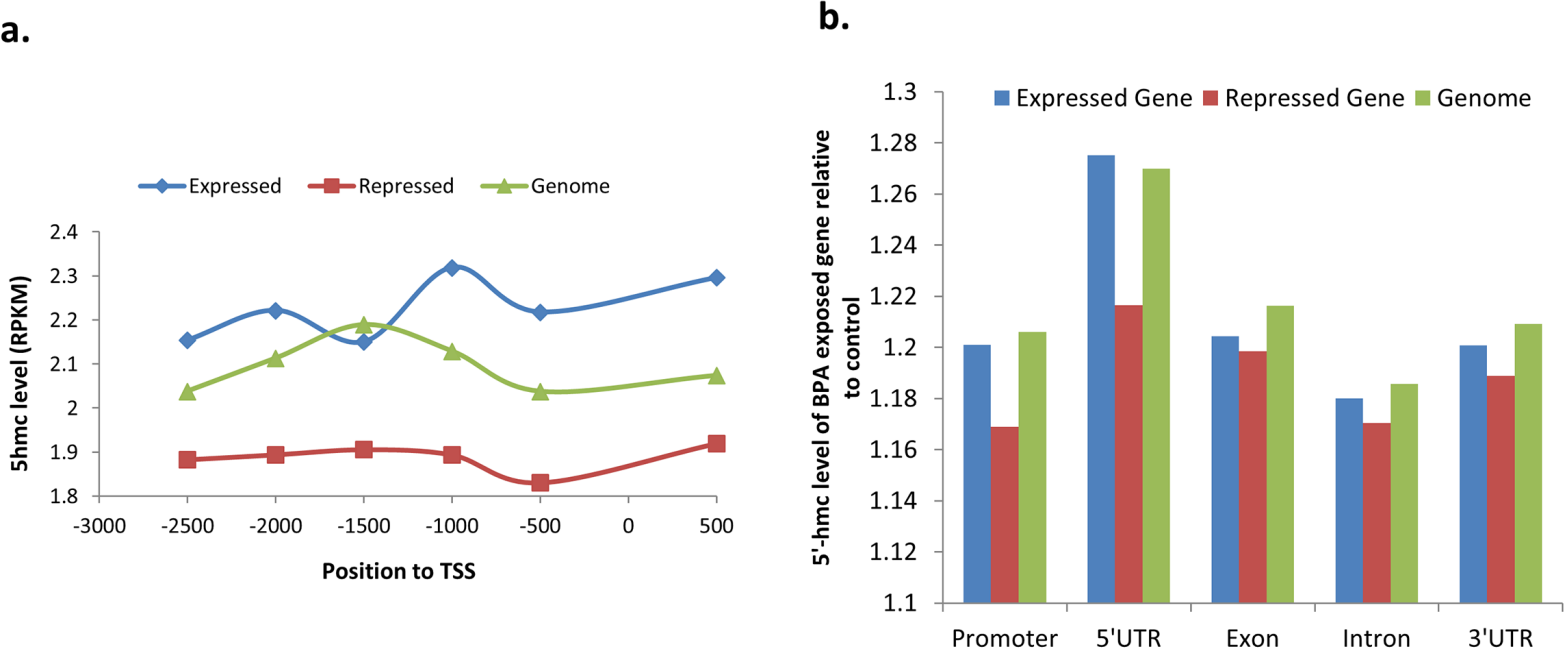
5hmc distribution and BPA affection in promoter and gene body of sperm expressed or repressed genes. **a**. Composition of 5hmc in promoter. **b**. Relative to control, the 5hmc level of each gene body in BPA-exposed sperm.

We analyzed the distribution of BPA-associated 5hmc in repeat regions, and found it showed a slight bias towards repeats. That is, 68.2% of repeats in the control while 80.2% of repeats in sperm with BPA exposure overlapped with 5hmc region (**Fig 2C** and **S5 Table**). We also examined the distribution of DhMRs on repetitive sequences. In general, LINEs were enriched with DhMRs (**Fig 2D**). Increased expression of LINE-1 is closely correlated with the decreased methylation in LINE-1 5’UTR [32]. Since the 5hmc level in 5’UTR was significantly improved in BPA-exposed sperm (**Fig 3C**), we speculated that the corresponding methylation level was decreased, thus BPA-related hyper-hMRs might active LINE-1, further damaging sperm DNA.

#### Imprinted genes associated with exposure to BPA

Imprinted genes show monoallelic or highly biased expression according to the parental origin of the allele and were controlled by DNA methylation differently in the parental germline [33]. A total of 210 human imprinted genes collected from NCBI and http://www.geneimprint.com/databases/ were analyzed in our study. We found that eight maternally expressed imprinted genes had their promoters hydroxymethylated in BPA-exposed sperm (**S6 Table**). Four of them (*FOXF1*, *SALL1*, *CDKN1C* and *HOXC4*) had been found hypomethylated in sperm with low motility [34] (**Table 1**), providing supportive evidence for our hypothesis that BPA-responsive DNA hydroxymethylation in the promoters of imprinted genes may have resulted in a low sperm motility that was previously observed in BPA-exposed men[4].

**Table 1:** Known sperm function associated genes affected by BPA.

Gene	Function	Express in Sperm	DhMRs	DhMRs Effect	Phenomenon
ATP5G3	Sperm motility	+	hypo-hMRs	down-regulation	affecting oxidative phosphorylation
NDUFV2	Sperm motility	+	hypo-hMRs	down-regulation	affecting oxidative phosphorylation
ACHE	Sperm motility	+	hyper-hMRs	overexpression	sperm impairments stress-related male infertility
ASAH1	Sperm motility embryo development	+	hypo-hMRs	down-regulation	
PPP2R3C	Sperm fertility	-	hyper-hMRs	up-regulation	globozoospermia
MLH1	DNA repair	-	hyper-hMRs	up-regulation	response to damaged sperm DNA
CHD2	DNA repair	+	hyper-hMRs	overexpression	DNA damage response and genome stability maintenance
SPATA12	DNA repair	+	hyper-hMRs	overexpression	DNA damage signaling
SPATA20	DNA repair	+	hyper-hMRs	overexpression	DNA damage signaling
RAD23B	sperm maturation	+	hypo-hMRs	down-regulation	
CUL3	male fertility	+	hypo-hMRs	down-regulation	
HSPA1L	sperm plasma membrane	+	hypo-hMRs	down-regulation	
UBAP2	Sperm fertility	+	hypo-hMRs	down-regulation	oligozoospermic infertile
TFPI2	embryo development Imprinted Gene	-	hyper-hMRs	up-regulation	affecting the normal early development
FOXF1	Sperm motility Imprinted Gene	-	hyper-hMRs	up-regulation	low motility
SALL1	embryo development Imprinted Gene	-	hyper-hMRs	up-regulation	low motility,disturb the embryonic stem cell differentiation
CDKN1C	embryo development Imprinted Gene	-	hyper-hMRs	up-regulation	low motility,resulted in embryonic growth retardation
HOXC4	Sperm motility	-	hyper-hMRs	up-regulation	low motility

#### Functional analysis of DhMRs

Compared with the controls, we found 8,670 hyper-hMRs and 940 hypo-hMRs in BPA-exposed sperm (**S7** and **S8 Tables**), affecting 2,008 genes (1,870 by hyper-hMRs and 274 by hypo-hMRs). These genes showed an uneven distribution on different chromosomes, with 11.8% of genes on chr18 (46/339) while only 2.6% of genes on sex chromosomes (29/1121 on chrX and 3/113 on chrY) affected by BPA exposure. As shown in **Fig 1B**, BPA exposure caused more 5hmc level increase (25–26%) in sex chromosomes than in autosomes (~19%), but DhMRs affected gene ratio on sex chromosomes were much lower than on autosomes. This might be caused by low gene density on sex chromosomes, as is known that human X chromosome has roughly half the gene density of human autosomes [35]. Interestingly, the hyper-hMRs affected genes were enriched with functional annotation terms related to cell adhesion (‘cell-cell adhesion’, ‘cell adhesion’ and ‘homophilic cell adhesion’, P value<1E-4), exocytosis (GO:0006887), cell migration, protein kinase A regulatory subunit binding, etc (**S9 Table**). It seemed that hyper-hMRs were associated with the expression of germ cell-interaction genes. In addition, the genes with the hyper-hMRs were enriched in seven pathways, mainly associated with nervous system (four pathways), development, cardiovascular diseases and signal transduction (**S10 Table**). Hypo-hMRs affected genes were enriched in 12 GO terms and two KEGG pathways, including oxidative phosphorylation (**S11 Table**).

### Analysis of association of DhMRs with sperm-expressed and repressed genes

The studies based on different techniques including microarray [36], SAGE [37] and RNA-seq [38, 39] have proved that human spermatozoa contain a complex repertoire of mRNAs and small RNAs, though their expression level is much lower than testes cells. By using RNA-seq, *Sendler et al*. identified 6,709 expressed genes (RPKM>1) in human sperm [39], with 726 genes considered as highly expressed genes (RPKM>50) in human sperm (**S12 Table**), while 523 abundantly expressed genes in testes (PRKM>25) were validated not expressed in sperm (RPKM<1) (**S13 Table**), which is defined as sperm-repressed genes.

Given 5hmC in the mouse genome is crucial for regulation of gene expression during the differentiation of spermatogenic cells[22], we speculated that it also modifies gene expression before human sperm maturation, considering that sperm are known to be transcriptionally silent. Therefore, we compared DhMRs in the genes with different expression in sperm. 678 genes sperm-expressed were observed to have hyper-hMRs, while 84 sperm-expressed genes were observed to have hypo-hMRs (**Fig 3**), indicating that 11.4% (762/6,709) of sperm-expressed genes have altered status of DNA hydroxymethylation as a result of BPA exposure. In contrast, status of DNA hydroxymethylation of only 6.85% (2,008/29,310) of total genes is responsible to BPA exposure, as mentioned above (**S5, S7 and S8 Tables**). Therefore, our analysis strongly suggests the presence of trend that BPA preferentially affects level of DNA hydroxymethylation of sperm-expressed genes (FDR = 2.05E-56).

Association of the 5hmc rates in the promoters and gene bodies with level of gene expression was next analyzed. We checked 5hmc level in promoter and gene body of sperm-expressed and repressed genes[39]. The 3.0k-bp fragment of each gene (from -2.5k to 0.5k, relative to TSS) was subdivided into six bins, with 500-bp for each bin to calculate the 5hmc level (**Fig 3A**). The 5hmc levels of promoter regions in sperm-expressed genes were significantly higher than in sperm-repressed genes (**Fig 3A**), indicating that enrichment of 5hmc in promoter plays a critical role for gene expression. This is in accordance with reported observations that 5hmC levels in the promoters are positively correlated with gene expression [22, 40].

We observed the presence of BPA-associated enrichment of 5hmc in every analyzed part of genes. Compared to sperm-expressed genes, the sperm-repressed genes showed a lower enrichment of 5hmc in gene bodies including 5’UTR, exon, intron and 3’UTR (**Fig 3B**), consistent with the above-mentioned observation that sperm-expressed genes are more sensitive to BPA exposure.

As mentioned above, BPA-associated alteration in 5hmc rate had no bias to the promoter region. However, we did find hyper-hMRs in the promoters of 58 genes. 24 out of the 58 genes harbored no detectable 5hmc in their promoters in the control, but significantly increased 5hmc level after BPA exposure (**S14 Table**). For example, the 5hmc rate in the promoter of AChE gene, which encodes acetylcholine hydrolyzing enzyme acetylcholinesterase, significantly raised from 0 in the control sperm to 5–6 (RPKM) in BPA-exposed sperm. *AChE* is sperm-expressed gene[39]. Recently, we detected an inverse correlation between the 5hmc rate in the promoter of *AChE* gene and sperm motility in the subfertile male population (submitted). Considering that sperm AChE activity was negatively related to the development of sperm motility during sperm maturation[41], the BPA-associated enrichment of 5hmc in the promoter of *AChE* gene suggests the presence of upregulation of *AChE* expression in the sperm from BPA-exposed men, eventually making an contribution to development of sperm with poor motility observed in BPA-exposed men[41].

We observed that 38 sperm-repressed genes [39] were enriched with hyper-hMRs, with seven of them in the promoter, 5’UTR, exon or TTS (**S13 Table**). One of them is *PPP2R3C*, which encodes Protein phosphatase 2A, regulatory subunit B, gamma. It had been found to be associated with globozoospermia [42].

BPA exposure was previously shown to cause DNA damage in human sperm[7] and murine sperm[43]. The transcripts of some DNA damage-associated genes are abundant in human sperm [39]. We found that their 5hmc levels are upregulated by BPA exposure (**S12 Table**). For example, a hyper-hMRs was present in the promoter of *CHD2* (chromodomain helicase DNA-binding protein 2), which was proposed to play an important role in the DNA damage response and genome stability maintenance [44]. SPATA12 (spermatogenesis associated protein 12) has been revealed interacting with CHD2 and playing a role in DNA damage signaling [45], and we also found the hyper-hMRs in the 1^st^ intron of *SPATA12* (**S12 Table**). Additionally, the hyper-hMRs was observed to be present in its 5’UTR of DNA mismatch repair gene *MLH1* (**S13 Table**). Taken together, the results suggest upregulated expression of the sperm-expressed genes via raised DNA hydroxymethylation participates in BPA-associated DNA damage and repairing during human spermatogenesis (**Table 1**).

On the contrary, nine sperm-expressed genes were observed to have hypo-hMRs in BPA-exposed sperm (**S12 Table**), and their expression might be accordingly down-regulated. *RAD23B*, highly expressed in the human adult testis and spermatozoa, plays an important role in spermatogenesis and sperm maturation by inhibiting Ub-dependent proteolysis [46]. *CUL3* (Cullin-3) was expressed in male germline and required for male fertility [47]. The testis-specific *HSPA1L* was associated with the human sperm plasma membrane during spermatogenesis and its expression peaks in spermatids [48]. The list of genes also includes *UBAP2* (ubiquitin associated protein 2) (**Table 1**), whose expression was reduced by 52-fold reduction expression in spermatozoa of oligozoospermic infertile men [49].

Our analysis identified hyper-hMRs in the gene bodies of 131 genes (**S15 Table**) whose expression was reported to be absent both in human testis and sperm[39]. One of them is *NOS*. Testicular NO synthase (NOS) activity is higher in old rats than younger one, and were proposed to be involved in NO-mediated intrinsic pathway signaling in age-related increase in germ-cell apoptosis in male rats[50]. Considering the hyper-hMRs in gene bodies is correlated with upregulated gene expression, therefore, BPA-associated hyper-hMR in the *NOS* gene could affect spermatogenesis via a raised rate in germ-cell apoptosis.

### Analysis of association of DhMRs with H3 methylation in sperm-expressed genes

**Fig 4:**
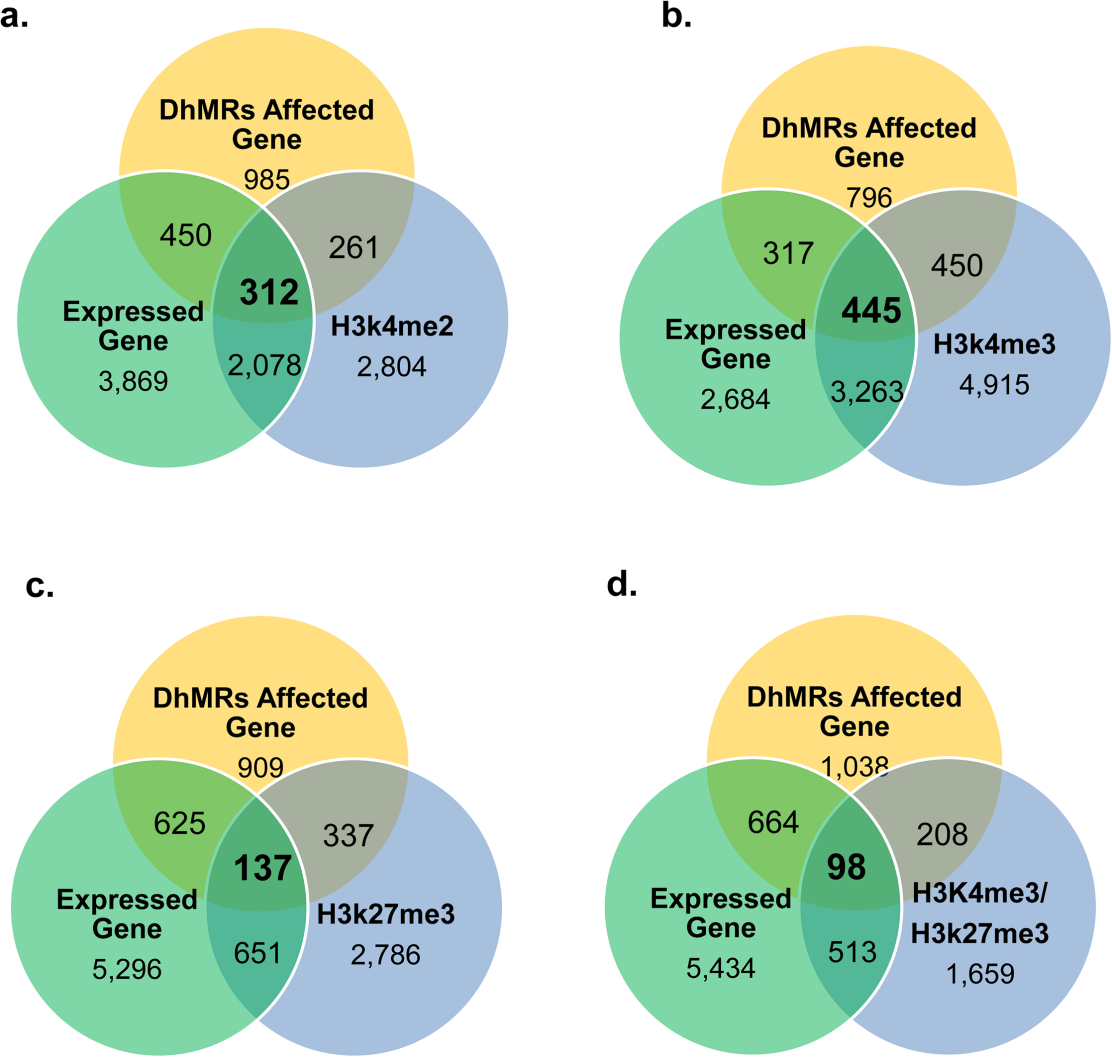
Relationship of 5hmc, histone binding and expression of sperm genes. **a.** relationship of H3K4me2 binding genes with DhMR genes and gene expression. **b.** relationship of H3K4me3 binding genes with DhMR genes and gene expression. **c.** relationship of H3K27me3 binding genes with DhMR genes and gene expression. **d.** relationship of H3K4me3/H3K27me3 binding genes with DhMR genes and gene expression.

The vast majority of mammalian sperm DNA is bound protamines, while nucleosome-binding sites constituting 2.9% of the paternal genome had been revealed in the human sperm genome [51]. We next analyzed whether BPA-associated alterations of DNA hydroxymethylation is associated with Histone H3 methylation. Based on ChIP-Seq analysis, two types of H3 Lysine 4 methylation (H3K4me3 and H3K4me2: two activating histone modification) were reported to exist in 5,455 and 9,073 genes, respectively in human sperm, while H3 Lysine 27 trimethylation (H3K27me 3: a repressive histone modification) is only present in 3,911 gene [52, 53]. Keeping in line with the reports, our analysis showed 43.9% (2,390/5,455) and 40.9% (3,708/9,073) of genes binding with H3K4me2 and H3K4me3, respectively, are expressed in sperm (**Fig 4A and 4B**). In contrast, only 20.1% (788/3911) of H3K27me3-binding genes is expressed in sperm (**Fig 4C**).

Association of DhMRs with the three types of H3 methylation was analyzed accordingly. Contrast to 6.85% (2,008/29,310) genes in sperm genome was associated with BPA exposure in sperm, a significantly higher percentage (from 9.86–12.1%) of H3 (bearing one of three types of lysine methylation) binding genes has DhMRs (**Fig 4A–4C**), indicating that H3 methylation raises efficiency of BPA-modifying DNA hydroxymethylation in sperm. Our analysis showed that 12.1% (474/3,911) of H3K27me3-binding genes were affected by DhMRs, while they were detected in 10.5% (573/5,455) H3K4me2- and 9.86% (895/9,073) of H3K4me3-binding genes, respectively (**Fig 4A–4C**). The difference is statistically significant (P-value between H3K4me2 and H3K27me3: 0.0038; P-value between H3K4me3 and H3K27me3: 2.529e-05), Similarly, in the 2,478 genes enriched both with H3K4me3 and H3K27me3 in human sperm[52] (or defined as bivalent genes), we observed that 12.3% (306/2,478) of bivalent genes harbored DhMRs (**Fig 4D)**. These results strongly suggested that BPA has a potential to preferentially raise level of 5hmc in the genes occupied with H3K27me3.

We further performed an analysis similar to the one above, focusing on whether the three types of H3 methylation differentially affect association of DhMRs with sperm profile of gene expression. Our analysis revealed that 44.6% (895/2,008) of the genes with BPA-responsive DhMRs bind H3K4me3, while only 28.5% (573/2,008), 23.6% (474/2,008) and 15.2% (306/2,008) of them binds with H3K4me2, H3K27me3 and H3K4me3/H3K27me3, respectively (The p-value between H3K4me3 and H3K4me2 was 2.2E-16, between H3K4me3 and H3K27me3 was 2.2E-163H) (**Fig 4A–4D**). The analysis demonstrated that the sperm-expressed genes are more sensitive to BPA for their alterations in DNA hydroxymethylation when they are enriched with H3K4me3 than other examined styles of H3 methylation.

### Analysis of BPA-associated 5hmc via qPCR

**Fig 5:**
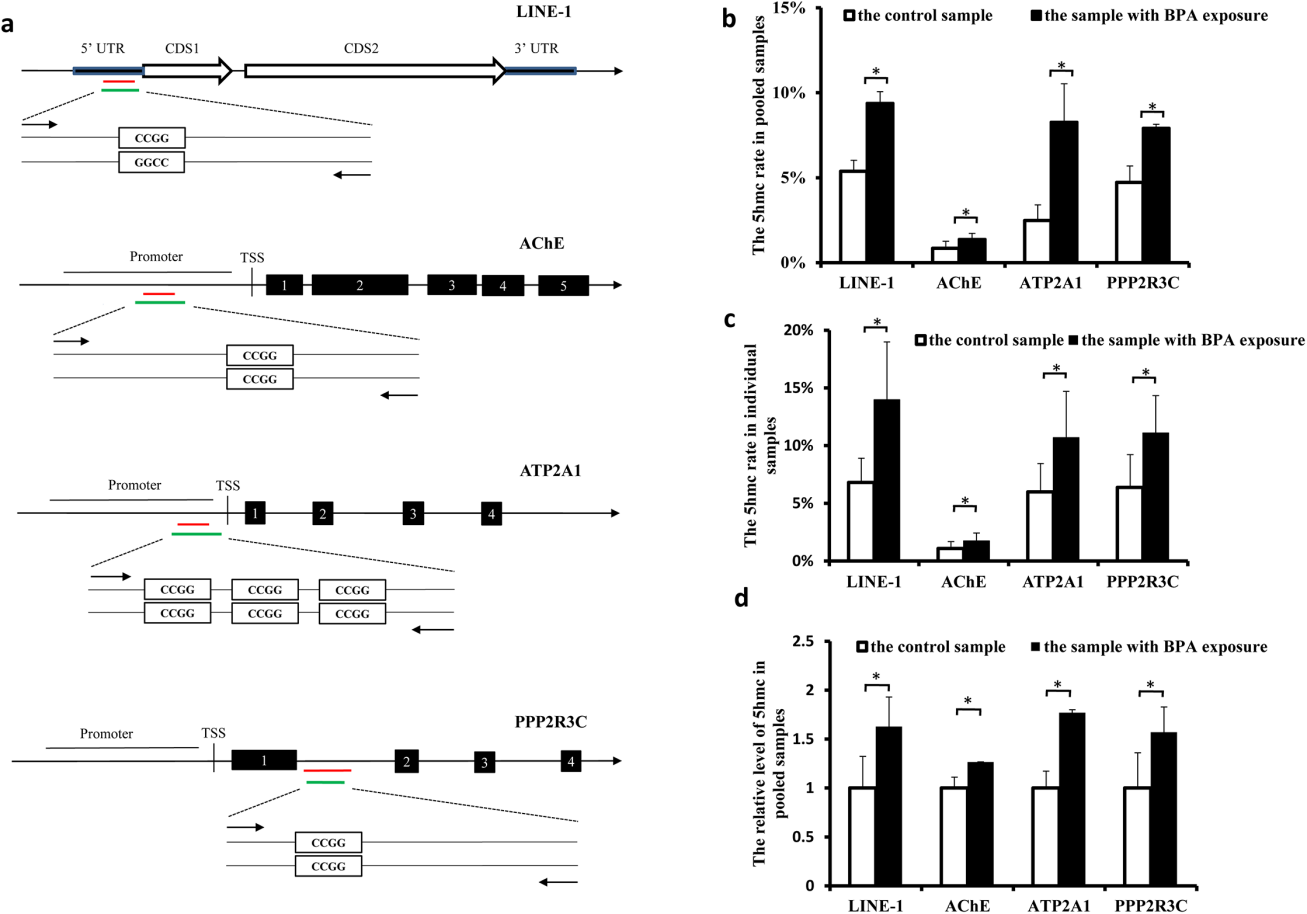
BPA raised 5hmc rate in sperm genes analyzed by qPCR. **a.** Schematic representation of location of the CCGG loci in the 5UTR /promoters/ gene body of LINE-1, AChE, ATP2A1 and PPP2R3C, respectively. The red line indicates the region where enrichment of BPA-associated 5hmc was detected through high-throughput sequencing, and it is 200-bp long in LINE-1, AChE, ATP2A1, and 300-bp long in PPP2R3C, respectively. The green line represents the location of qPCR product. **b and d.** The 5hmc rates of four genes were measured using the pooled sperm samples from BPA-exposed men and the control men. The sperm DNA was treated with glucosylation followed by CCGG loci-dependent MspI/HpaII digestion (b), or was immuno-precipitated with the 5hmc antibody (d), and then used as the template of qPCR. The data were obtained from at least three independent experiments, and the values are shown as the mean ± SEM; * p< 0.05. **c.** The 5hmc rates were measured using individual samples. Five BPA-exposed samples and five control samples were randomly chosen for measurement of 5hmc rate. The values are shown as the mean ± SEM; * p< 0.05.

We also compared the rates of 5hmc in sperm from men exposed to BPA with those from the men without significant BPA exposure. Four genes (*AChE*, *ATP2A1*, *PPP2R3C* and *LINE-1*) were detected to have hyper-DhMRs in the regions of promoter, intron or 5’UTR, as indicated in **Fig 5A**, and were selected for the analysis.

The 5hmC rates in the CpG sites were measured by qPCR after sperm DNA was applied to treatment with glucosylation followed by CCGG loci-dependent MspI/HpaII digestion[54]. We first used the pooled DNA samples for the measurement. qPCR assays across the selected CCGG sites showed a varied ranges of 5hmC rates, i.e., from 0.85% to 5.38% in the four genes, with the lowest rate detected in the *AChE* promoter. Moreover, we observed significant higher 5hmc rates in all the four genes in sperm from men exposed to BPA than the control (**Fig 5B**).

Considering the variations of 5hmc rates among different individual samples were not reflected in the pooled samples-based analysis, we repeated the above measurement in five randomly-selected sperm samples with BPA exposure and five randomly-selected control samples (**Fig 5C**). Again, our results showed BPA exposure raised the 5hmc rates in these genes with an efficiency comparable to that observed in the pooled samples.

Given our data were generated based on hMeDIP assay in the present study, we finally compared levels of 5hmc in these genes in BPA-exposed samples with the control by using the pooled DNA samples that were immuno-precipitated with the 5hmc antibody. As expected, a BPA-associated rise in the 5hmc rate was detected in all the four genes (**Fig 5D**). Importantly, by using two different techniques, we observed BPA exposure raised the 5hmc rate with a similar efficiency in the four genes, respectively (**Fig 5B and 5D**).

## Discussion

We reported in the present study that global level of DNA hydroxymethylation is higher by 19.6% in sperm from BPA-exposed men than from men without occupational exposure to BPA. The BPA-responsive alteration in DNA hydroxymethylation is characterized by the following two features: (1), 5hmc rates in sex chromosomes are more sensitive than autosomes to BPA exposure. It is currently unknown whether it is associated with BPA’s weak estrogen activity; and (2), LINES has been identified as the repetitive elements that are more sensitive to BPA exposure than any other genes and the repetitive elements. Of the genes affected by BPA exposure, the retrotransposons are particularly intriguing. They are thought to be maintained in a transcriptionally silent state by DNA methylation[55], but they are activated via BPA-induced DNA demethylation [56]. Positive correlation between expression and 5hmC density of retrotransposons during spermatogenesis was recently reported, highlighting the important role of 5hmc in regulating of retrotransposon activity [22]. It is noteworthy, therefore, that BPA raises 5hmc rates in retrotransposons including LINE-1 of human sperm, consistent with the hypothesis that BPA-responsive hypomethylation of LINE-1 detected in human sperm[19] is mediated by enhanced DNA hydroxymethylation. Given more than 100 LINE-1-mediated insertions which result in genetic diseases have been reported [57] and derepression of LINE-1 along with complete male sterility is linked to azoospermia [58], upregulated 5hmc rate in LINE-1 likely raises genomic instability, eventually making a contribution to reduced sperm concentration in the men exposed to BPA.

Besides the repetitive elements, approximately 2,000 sperm genes were observed to have changed their 5hmc rates as a result of BPA exposure. In a rational manner, we hypothesized that BPA exposure modifies the epigenetic setting of genes at different stages of spermatogenesis, and altered expression of some of these genes impairs spermatogenensis via distinct mechanisms. It has been recently recognized that 5hmC distribution undergoes an interesting dynamic change in the entire genome during mouse spermatogenesis[22]. Consistent with the report, levels of expression of Tet1-3 are positively correlated with sperm quality [59]. Importantly, concerning ICSI-outcome, the lowest levels of TET1-3 mRNAs in sperm were found in the non-pregnant group, while increased TET2 in sperm was significantly associated with pregnancy[59]. Globally altered expression of 5hmc has been reported to abnormal sperm of humans [60]. These reports indicated that an optimal pattern of genomic DNA hydroxymethylation is pivotal for maintenance of seminal quality and male fertility. Thus, genome-wide rise of the 5hmC marker is a new epigenetic feature of reproductive toxicity of BPA, and could be potentially regarded as a biomarker for BPA exposure.

The mechanism(s) underlying globally raised 5hmC in sperm responding to BPA exposure needs to be investigated and understood. The global level of 5hmC is reduced as male mouse meiosis starts [22], and BPA exposure disrupted meiosis progression [61]. These results suggest the BPA represses expression of TET1-3, which are the key regulators of global level of 5hmc during spermatogenesis. The mechanistic process may be investigated using *in vitro* approaches in future studies. However, multiple factors may be involved in the control of sperm genome-wide DNA hydroxymethylation in sperm, as suggested by an opposite changes in level of 5hmc [62] and TETs [59] was observed in sperm from aging men. Methyl-CpG-binding protein 2 (MeCP2) has been identified as the major 5hmC-binding protein in the brain [63]. Its expression also improves the development of mouse somatic cell nuclear transfer embryos, via a 5hmc-upregulating mechanism[64]. Interestingly, BPA exposure obviously upregulated expression of MeCP2, given abundance of MeCP2-expressing neurons significantly increased[65]. Therefore, it may be speculated that BPA raises genome-wide level of sperm 5hmc in the way depending on MeCP2. It is worthy for future study.

Our analysis demonstrated that the 5hmc levels in gene bodies and promoters were correlated with their levels of expression in human sperm, respectively, consistent with the previously reported observation that the 5hmc level positively regulated gene expression in eight consecutive types of mouse spermatogenic cells[22]. In a rational manner, we speculate that BPA exposure modifies the epigenetic setting of 2,008 genes at different stages of spermatogenesis. We observed that BPA preferentially affects the 5hmc levels of sperm-expressed genes, and such a modification probably depends on H3 methylation, given that it raised BPA-altering 5hmc in sperm. Additionally, studies have revealed a close association of 5hmc with histone modifications. For example, enrichment of 5hmc exists in the genes bound with active (H3K4me2 and 3) and repressive (H3K27me3) H3 modification [66]. Via the analysis based on co-immunolabeling of 5hmC and H3K27me3, Haffner et al. also reported that global level of 5hmc and H3K27me3 are tightly co-regulated during hierarchical differentiation in adult tissues [67]. In the present study, we observed that BPA has a potential to preferentially raise the enrichment of 5hmc in the genes occupied with H3K27me3. Enhancer of Zeste Homolog 2 (EZH2) is known to be the catalytic subunit of the polycomb repressive complex 2 (PRC2), a histone methyltransferase catalyzing trimethylation of H3K27. The distribution of Ezh2 was detected in spermatogonial stem cells [68] and the round spermatids [69]. Although it remains unknown whether BPA affects EZH2 expression during spermatogenesis, it did so in both cultured breast cancer cells and mouse mammary [70, 71]. Taken together, these results suggest BPA raises 5hmc rates during human spermatogenesis in the way dependent on EZH2-based H3K27me3.

Altered pattern of DNA methylation in sperm has been proposed to play an important role in a transgenerational toxic effect caused by paternal exposure of BPA [17, 72] and other environmental factors. Reprogramming of the paternal genome upon fertilization involves genome-wide oxidation of 5-methylcytosine-mediated by manteral TET3 [41]. Aberration in status of paternal DNA hydroxymethylation has been shown to be linked to poor preimplantation embryo development [73, 74]. For example, human [60] and mouse [22] round spermatids have a globally-altered pattern of 5hmc, compared with mature sperm, while active DNA demethylation normal-sized embryos were detected in a much reduced rate in the embryo with injection of round spermatid[73]. These reports suggest a close relationship between paternal alteration of 5hmc and impaired development [75]. Accordingly, our present study also indicates a potential risk that paternal alteration in DNA hydroxymethylation participates in a transgenerational toxic effect of BPA.

It should be noted that urine BPA level in the BPA-exposed men in the present study is quite high compared with those who were exposed through environmental sources. Further investigation is needed to examine whether the above observed association also exists at lower BPA exposure levels.

## Conclusions

We reported a genome-wide rise in 5hmc rate in the sperm from BPA-exposed men in the present study. To our knowledge, this is the first report regarding the effect of BPA on DNA hydroxymethylation in human tissue. Our results suggest that BPA may affect gene expression and reduce genomic stability via altered rate of 5hmc, eventually resulting a previously reported low concentration and motility of sperm observed in BPA-exposed men [4]. Therefore, our study provides new evidence for a potential underlying mechanism of sperm toxicity of BPA exposure.

## Materials and methods

### Ethics statement

The study was reviewed and approved by the ethics committee board of Shanghai Institute of Planned Parenthood Research (IRB00008297). All participants gave written informed consent before participation in the study.

### Semen samples

Collection of semen samples from the workers with BPA exposure and from the control men without significant BPA exposure was previously described [4]. The semen samples of exposed group were from 30 workers with an average age of 33.6 years, while those of the unexposed group were from 26 workers with an average age of 34.4 years. To be eligible as a subjects in the present study, the exposed workers had to be: 1) working in a BPA manufacturer or epoxy resin manufacturer; 2)aged between 20 to 49 years; 3) with a job title of packaging, chemical reaction, or material feeding, which were supposed to be exposed to high doses of BPA. While the unexposed workers are recruited if they were: 1) working in a factory in the same city as the exposed factory with no known occupational BPA exposure, nor known reproductive toxicants; 2) aged between 20 to 49 years. The median of urine BPA levels in the exposed and unexposed group were 251.33 ug/g creatine and 0 ug/g creatine, respectively. The percentages of smoking for the exposed and unexposed group were 43.3% and 42.3%.

### hMeDIP

hmeDIP assays were performed on sperm DNA as described for 5mC as previously described by Mohn et al. [76] using commercial antibodies specific to 5hmC (Active Motif). Genomic DNA was extracted from the sperm from 30 BPA-exposed men and 26 controls, respectively. The BPA-exposed DNA and the control DNA were generated by the pooling of each sample with an equal amount of DNA. The pooled DNA was then sonicated to an average size of 200bp (range 100-500bp). DNA fragments were end-repaired, A-tailed and custom TruSeq adapters (non-methylated) were ligated. 1μg of adapter-ligated DNA was denatured and incubated with 1μl of 5hmC antibody at 4°C overnight. Antibody-DNA complexes were captured by protein A/G beads. The immunoprecipitated DNA was purified and then applied for sequencing analysis.

### DNA hydroxymethylomes

The genome-wide maps of 5hmC were produced by performing hydroxymethyl-DNA immunoprecipitation followed by massively parallel sequencing with an Illumina Genome Analyzer (hMeDIP-seq). A total of 35,995,280 reads of 51 bp was obtained from control, and 44,263,854 reads of 51 bp was obtained from sperm with BPA exposure. Sequence quality control was performed using FASTQC.

SAMtools [77] was used to remove reads with low alignment score (Q < 20). Alignment to reference genome hg19 build downloaded from UCSC was performed using BWA (v0.5.8) [78] with default parameters. Then hMeDIP-seq data were processed using R-based Package ‘MEDIPS’ [28] with genomic window size set to 100 bp. For each sample, the aligned reads count at each window is transformed into reads per kilobase per million (RPKM) format. The region was defined as being covered if any part of the region was covered by a minimum of one sequencing reads. The saturation analyses resulted in genome-wide coverage saturation of 0.93 for control and 0.92 for case (**S2 Fig**).

Moreover, we tested the enrichment of CpG-rich short reads derived from the immunoprecipitation step, and found a relative enrichment for CpG-rich short reads from the control sample (1.05) and case sample (1.03) compared with the reference genome. Unexpectedly, the relative CpG enrichment is close to one for both samples (**S16 Table**).

### DhMR analysis

P-values were calculated using edgeR by comparing the RPKM of the sperm with BPA exposure and control samples within each of the 100-bp windows. Differentially 5hmc regions (DhMRs) were identified by filtering for windows associated with a P-value ≤0.005. Then GO and pathway enrichment analysis of genes affected by hyper-hMRs or hypo-hMRs were separately performed based on EntrezID using DAVID [79].

## Supporting information

S1 Fig CpG coverage analysis. The figure shows the results of hMeDIPS coverage analysis for control (**a**) and case (**b**).
(TIF)

S2 Fig Saturation analysis. The figure shows the results of the saturation analysis of the MEDIPS package analyzing hMeDIP-seq data from control (**a**) and case (**b**).
(TIF)

S1 Table Distribution of 5hmc in gene body. (XLS)

S2 Table Distribution of CpG islands covered by hMeDIP reads. (XLS)

S3 Table Distribution of promoter region covered by hMeDIP reads. (XLS)

S4 Table Differentially hydroxymethylated regions (DhMRs) between BPA-exposed men and controls. (XLS)

S5 Table Distribution of hydroxymethylated regions in repeats. (XLS)

S6 Table 5hmc level in promoter of human imprinted genes. (XLS)

S7 Table Genes affected by hyper-hMRs in BPA-exposed population. (XLS)

S8 Table Genes affected by hypo-hMRs in BPA-exposed population. (XLS)

S9 Table Enrichment analysis of hyper-hMRs affected genes. (XLS)

S10 Table Pathway enrichment analysis of hyper-hMRs affected genes. (XLS)

S11 Table Enrichment analysis of hypo-hMRs affected genes. (XLS)

S12 Table Highly expressed genes in sperm was affected by hMRs. (XLS)

S13 Table Lowly expressed genes in sperm was affected by hyper-hMRs. (XLS)

S14 Table Genes with promoter affected by DhMRs in the BPA-exposed population. (XLS)

S15 Table Genes with gene body affected by hyper-hMRs in the BPA-exposed population. (XLS)

S16 Table CpG enrichment analysis of the MEDIPS package. (XLS)
