# Supplementary figures and images for: Genome-wide alteration in DNA hydroxymethylation in the sperm from bisphenol A-exposed men
Source: PLoS One. 2017 Jun 5;12(6):e0178535. doi: 10.1371/journal.pone.0178535 (PMC5459435; doi:10.1371/journal.pone.0178535)

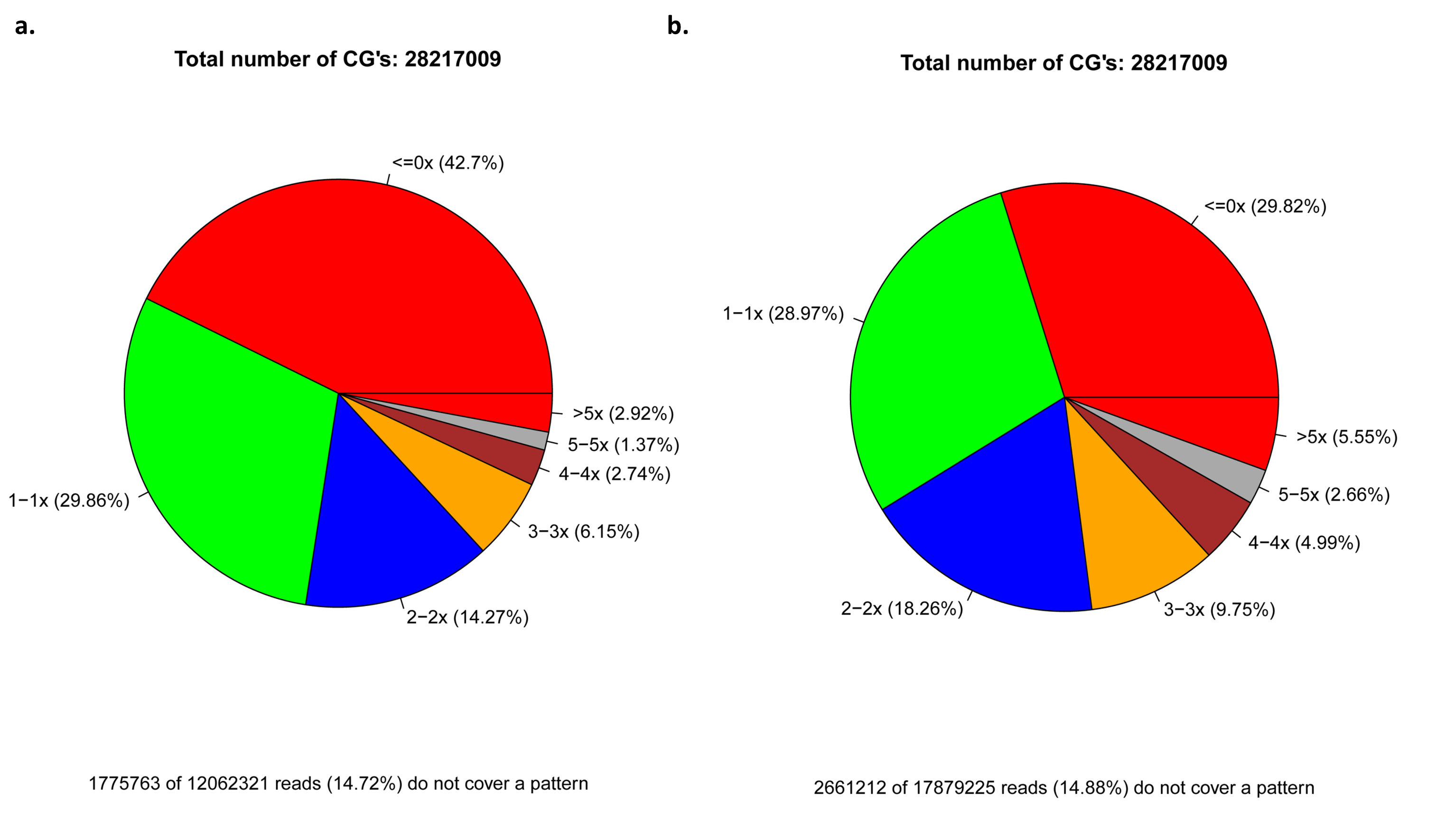

Supplement: S1 Fig — The figure shows the results of hMeDIPS coverage analysis for control (a) and case (b). (TIF) [file pone.0178535.s001.tif]

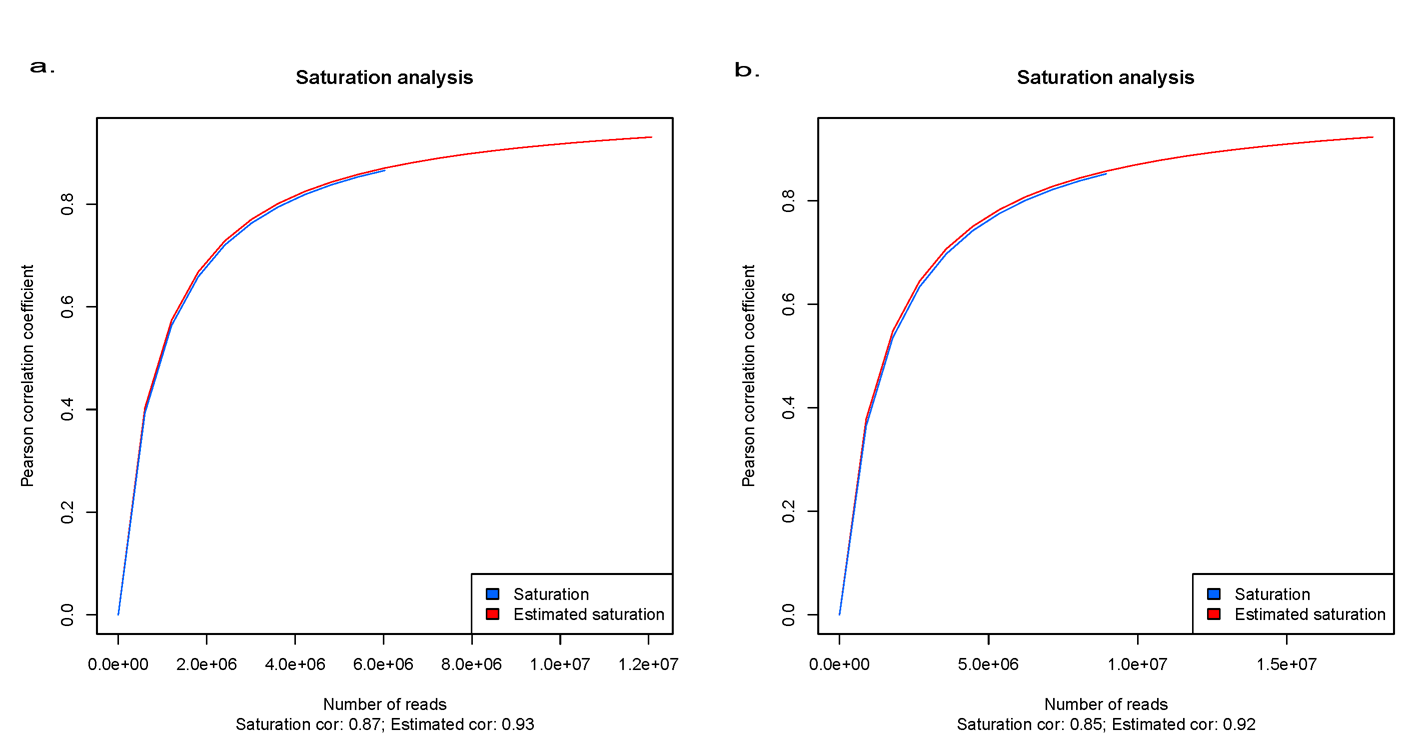

Supplement: S2 Fig — The figure shows the results of the saturation analysis of the MEDIPS package analyzing hMeDIP-seq data from control (a) and case (b). (TIF) [file pone.0178535.s002.tif]
